# The role of ATF3 in precision medicine of brain arteriovenous malformation: based on endothelial cell proliferation

**DOI:** 10.3389/fimmu.2025.1567970

**Published:** 2025-06-27

**Authors:** Jiwei Ma, Lei Meng, Jiyu Ji, Xiangyang Wang, Nan Wu, Lei Sun, Shupeng Zhao

**Affiliations:** ^1^ Department of Neurosurgery, The First Affiliated Hospital of Xinxiang Medical University, Weihui, Henan, China; ^2^ Department of Neurology, The First Affiliated Hospital of Xinxiang Medical University, Weihui, Henan, China; ^3^ Department of Pharmacy, The First Affiliated Hospital of Xinxiang Medical University, Weihui, Henan, China; ^4^ Shandong University of Traditional Chinese Medicine, Jinan, Shandong, China

**Keywords:** brain arteriovenous malformation, single-cell sequencing, endothelial cells, ATF3, experiment validation, precision medicine

## Abstract

**Background:**

Brain arteriovenous malformation (BAVM) is a destructive high-flow vascular abnormality that can lead to various cerebral hemodynamic disorders. The incidence of BAVM has risen significantly in recent years, yet treatment options remain limited. Endothelial cells (ECs) have been proved to be one of the key factors leading to abnormal cerebrovascular structure. Therefore, it is important to explore the pathogenesis of the disease and develop new treatment strategies. With the rapid advancement of single-cell sequencing (scRNA-seq) and the integration of multi-omics data offers a novel perspective for precision medicine.

**Methods:**

We first analyzed scRNA-seq data from the GEO database. We used monocle2, CytoTRACE, and slingshot to perform pseudotime trajectory analysis on ECs. CellChat was used to analyze cell-cell communication in BAVM, and pySCENIC was used to analyze related transcription factors (TFs). Finally, transfection, CCK-8, RT-qPCR, Transwell, EdU, tube formation, and other commonly used experiments were conducted to further validate the effects of key TFs on ECs intervention.

**Results:**

scRNA-seq analysis showed that ECs in BAVM had significant specificity. C0 subpopulation was the key subpopulation, showing strong proliferation and differentiation ability. This study emphasized that the midkine(MK, MDK)signaling pathway was a significant signaling pathway. Heparin-binding growth factor midkine was a secreted protein with a molecular weight of 13 kDa. Studies had shown that it can promote endothelial cell proliferation and lead to angiogenesis. Then, the C0 subpopulation was also associated with a variety of TFs, among which ATF3 played a key role in the pathogenesis of BAVM. The possibility of ATF3 affecting the progression of BAVM was verified by cell experiments.

**Conclusion:**

This study employed scRNA-seq and multi-omics analysis to elucidate the pathogenesis of BAVM, uncovering the key role of ATF3 in ECs proliferation. Targeting ATF3 provided a new possibility for the treatment of BAVM and also made up for the blank of ATF3 in the exploration of BAVM. This study provided a theoretical basis for the personalized treatment of BAVM and demonstrated the potential of scRNA-seq and multi-omics methods in advancing precision medicine.

## Introduction

Brain arteriovenous malformation (BAVM) (1) is traditionally considered to be a congenital cerebrovascular malformation, which originates from abnormal embryonic cerebrovascular development. It occurs due to the absence of capillaries between the affected brain arteries and veins, causing direct connections between them. This disrupts the normal blood flow pattern, allowing blood to bypass the capillaries and flow directly from the arteries into the veins, leading to a series of hemodynamic disturbances in the brain (2). However, in recent years, more and more clinical reports (3, 4) have shown that BAVM may have acquired characteristics, such as recurrence in lesions treated with angiography and radiotherapy after surgical resection. BAVM predominantly affects young and middle-aged individuals, with the majority of cases occurring between the ages of 20 and 40. Clinically, it often manifests through recurrent intracranial hemorrhages, seizures, headaches, and so on. Research indicates that 65% of BAVM patients exhibit symptoms of cerebral hemorrhage, with the risk of bleeding markedly increasing after the initial hemorrhage. Approximately 25% of patients experience a recurrence within four years (5). In addition to the above clinical symptoms, BAVM also seriously affects the quality of life of patients. With the passage of time, patients often have neurological impairment and mental and psychological problems, which cause great inconvenience to their daily lives.

Research suggests that the vascular endothelial cells (ECs) are one of the key factors contributing to the formation of BAVM (6, 7). ECs are distributed throughout the circulatory system, and new angiogenesis is related to the differentiation, proliferation, migration, adhesion and permeability of ECs (8, 9). They gradually form new capillaries, arteries, and veins from the initial vasculature, expanding into a vascular network. Among them, the differentiation of arteries and veins is related to genetic factors, mechanical stress, inflammatory responses, metabolism, and other stimulating factors (10). Moreover, cerebral vascular ECs also express multiple signaling pathways and TFs related to BAVM, among which the Notch and Wnt signaling pathways are key factors in the disease progression (11, 12). The Notch signaling pathway (Notch1 and Notch4) primarily regulates angiogenesis, EC proliferation, and artery-vein differentiation, playing a role in the pathogenesis of human AVM (13). Both Notch1 and Notch4 can induce BAVM (14, 15), with Notch4 being more prominent in ECs. The Wnt signaling pathway, associated with embryonic angiogenesis, promotes blood vessel formation when activated and is considered a risk factor for inducing BAVM (11). KRAS is very important in KRAS/MAPK/ERK signal transduction (16), and mutant KRAS can overactivate the MAPK/ERK pathway to promote the growth and proliferation of ECs (17, 18). Inflammation, hypoxia, and compromised vascular wall integrity or excessive vascular growth are also significant contributing factors to BAVM.

At present, the diagnosis of BAVM mainly depends on imaging examinations, including CT, MRI, and digital subtraction angiography (DSA). In terms of clinical classification, BAVM can be divided into familial hereditary and sporadic types (1, 19–21) according to different mutation patterns. The most common familial hereditary BAVMs are hereditary hemorrhagic telangiectasia (HHT) and capillary malformation-arteriovenous malformation (CM-AVM), both of which are autosomal dominant genetic diseases (22). Among them, the occurrence of HHT is related to genes such as ENG, ALK1, SMAD4, and GDF2 (23), while CM-AVM may be related to RASA1 and EPHB4 mutations (24). It is worth noting that sporadic BAVM accounts for about 95% (21) of the total number of cases, and most of the somatic KRAS mutations (25, 26) can be detected, so this study is mainly aimed at sporadic BAVM patients (17, 27). BAVM mainly adopts three treatment methods: microsurgery, stereotactic radiotherapy and interventional therapy, in order to control the risk of the disease (28, 29). However, due to an incomplete understanding of the mechanisms behind BAVM development and progression, no suitable drug therapies have been developed. Furthermore, surgical treatment involves considerable risks and has limitations in terms of safety. The overall cure rate for cerebral arteriovenous malformations is 46%, and they are prone to recurrence. Approximately 25% of patients will experience recurrence within the first year after intervention (30). Therefore, identifying risk factors to achieve precision treatment is urgently needed.

Traditional cell sequencing methods cannot deeply explore the cell heterogeneity in disease progression, especially it is difficult to accurately reveal the functional specificity and dynamic evolution of specific cell subpopulations. In contrast, scRNA-seq technology (31–36), as a powerful tool for studying diseases, enables genome sequencing and analysis of single cells. This technology allows detailed analysis of cell heterogeneity at the single-cell level, so it has been widely used in the study of various diseases, providing important support for in-depth understanding of disease mechanisms and the development of precise treatment method (25). Although relevant analyses of BAVM exist, there remains a significant gap in understanding the mechanisms of BAVM development and identifying prognostic biomarkers and therapeutic targets. To develop safer and more effective treatment methods for clinical practice, further research into BAVM is essential.

Therefore, we analyzed the scRNA-seq data of BAVM patients from the GEO database to reveal the disease mechanism and treatment strategies at single-cell resolution. We elucidated the transcriptomic features of ECs, identifying the C0 *TSHZ2* + ECs as a pivotal subpopulation due to their pronounced specificity in cell cycle and developmental trajectories. Subsequently, we examined the developmental trajectory of C0 *TSHZ2*+ ECs, conducted functional enrichment analyses, and evaluated relevant transcription factors (TFs). Our main goal is to further explore the complex interaction mechanism between ECs and BAVM and to clarify its key role in the occurrence and development of diseases. At the same time, we are committed to screening and verifying potential therapeutic targets (26, 37). As the core hub of gene expression regulation, TFs play a key role in cell proliferation, cell cycle transition, embryonic development and differentiation, and disease progression (38). TFs can be used as a starting point to trace to the source of regulation and can also achieve the overall grasp and effective intervention of the entire disease by controlling the transcription of multiple downstream target genes. For cell communication, TFs can participate in the regulation of multiple signaling pathways, and multiple pathways work together to act on disease progression (39). In addition, TFs play a crucial role in the pathological process of BAVM. Several studies have confirmed that TFs are involved in the regulation of the occurrence and development of BAVM (40, 41).

Therefore, we focus on selecting effective targets for BAVM from the perspective of TFs. The selected targets will be evaluated by cell experiments (such as EDU, CCK-8, RT-qPCR and tube formation experiments) to show their specific expression in cell proliferation, migration, and other processes. Finally, it will provide a scientific basis for the accurate treatment and effective clinical intervention of the disease.

## Methods

### Source of single cell dataset

All data for this study were sourced from the Gene Expression Omnibus (GEO) database (https://www.ncbi.nlm.nih.gov/geo/) (42, 43) under the accession number GSE256490. The single-cell dataset included samples of three BAVM patients and six temporal lobe (TL) patients (GSM8101569-GSM8101577) (44). The data were derived from public databases and do not require ethical review. However, it should be noted that this study failed to obtain detailed clinical information from the original data.

### scRNA-seq data processing and analysis

We began by utilizing the R software (version 4.2.0) and Seurat (version 4.1.1) software package to integrate scRNA-seq data and conduct quality control (QC) on the raw data (45–47). In order to obtain high-quality cells, a set of QC standards was specified, including: (1) 300 < nFeature_RNA < 7,500, (2) 500 < nCount_RNA < 10,000, (3) mitochondrial gene expression below 25% and red blood cell values below 5%, and (4) a unique molecular identifier (UMI) count greater than 1,000. Cells failing to meet these QC criteria were excluded, and we used the “DoubletFinder” R package (25, 48, 49) to filter out doublet cells from each sample individually (50).

The application of the “NormalizeData” function to normalize the data (51–54). The highly variable genes (HVGs) were screened by “FindVariableFeatures” (55–57). Then, the “ScaleData” function was used to standardize the top 2000 HVGs, which were subjected to principal component analysis (PCA) (58–62). To address batch effects, we processed the data using the Harmony package. In addition, we would use the first 30 principal components (PCs) selected after RunPCA dimensionality reduction for analysis and visualization through Uniform Manifold Approximation and Projection (UMAP) (63). Cell markers were acquired from the CellMarker website (64) and relevant literature, and cell clusters were identified based on these cell markers.

### Enrichment analysis of differentially expressed genes and AUCell analysis of cell subpopulations

The FindAllMarker function was employed to identify DEGs for each cell type in BAVM (64). DEGs accounted for more than 25% of all cell types. We then utilized ClusterProfiler to conduct enrichment analysis on the DEGs of each cell type in BAVM, focusing on Gene Ontology (GO) enrichment analysis (65–71), which included enrichment of molecular functions (MF), cellular components (CC), biological processes (BP), and relevant signaling pathways (72). A P-value < 0.05, | log2 (FoldChange) | > 1, and FDR < 0.05 were considered statistically significant. AUCell values were primarily used to identify cells with active gene expression.

### Pseudotime analysis of ECs

We employed CytoTRACE, Monocle2 (73), and Slingshot (74) to infer pseudotime trajectory of ECs. CytoTRACE was a computational tool designed to infer the relative differentiation status of scRNA-seq data. It could predict cell stemness. Monocle2 was a novel unsupervised algorithm that ordered cells along a differentiation process. It was used to infer the cell trajectory of ECs. Slingshot analyzed the pseudotime trajectory of ECs. By selecting DEGs between clusters for PCA-based dimensionality reduction and visualizing the pseudotemporal dynamics of different subpopulations on UMAP plots.

### Cell-cell communication analysis

We used the and CellChat (75, 76) to analyze cell-cell communication networks. We collected the main input and output signals of each cell type and depicted them. In addition, we also analyzed the intercellular signaling pathway (77).

### SCENIC analysis

SCENIC (78) was a tool that constructed gene regulatory networks from scRNA-seq data and can identify cellular activity states. We used the pySCENIC package (version 0.10.0) in Python (version 3.7) to construct gene networks and regulatory factor activity (79). The AUcell value was calculated to evaluate the activity of regulatory factors in cells, and then the top TFs were selected.

### Cell cultures

Primary human umbilical vein endothelial cells (HUVECs) were obtained from the American Type Culture Collection (ATCC) (80, 81) and cultured in endothelial cell medium (ECM) supplemented with 10% fetal bovine serum (Gibco BRL, USA) and 1% streptomycin/penicillin. The cells were cultured under controlled conditions at 37°C, with 5% CO_2_ and 95% humidity. The density of HUVEC was more than 80%, and 5 × 10^5^ cells were seeded into culture dishes within 48–72 h to promote the next experimental study.

### Transfection

Inhibition of ATF3 was achieved through siRNA constructs, with transfection conducted using Lipofectamine 3000 RNAiMAX (Invitrogen, USA). A negative control (siNC) and knockdown variants (si-ATF3–1 and si-ATF3-2) were introduced.

### The CCK-8 assay

Cells (3 × 10³ per well) were seeded in 96-well plates and cultured for 24 hours. Subsequently, 10 μL of CCK-8 reagent (A311-01, Vazyme) was added per well, and the plate was incubated at 37°C, 5% CO_2_, and 95% humidity for 2 hours. Cell viability was assessed by measuring the absorbance at 450 nm at time points of 0, 24, 48, 72, and 96 hours using a microplate reader (A33978, Thermo) (82).

### The real-time polymerase chain reaction Analysis

Total RNA was extracted from the HUVEC cell lines using TRIzol reagent and reverse-transcribed with the PrimeScript™ RT reagent kit (Vazyme, R232-01) (83–85). The RT-qPCR (84) was performed using the SYBR Green Kit (TaKaRa Biotechnology, Dalian, China), with GAPDH as the internal reference. See **Supplementary Material 1** for the specific primer order.

### Western blotting

The transfected cells were treated with RIPA lysis buffer, and then the cell lysate was centrifuged at 12,000 rpm for 15 minutes (4°C) to remove cell debris, and the supernatant was collected for later use (86, 87). The protein was separated by SDS-PAGE electrophoresis according to the difference in protein molecular weight, and the separated protein was transferred to a PVDF membrane. In order to block the non-specific binding site, 5% bovine serum albumin (BSA) was used to block at room temperature for 1.5 hours. Subsequently, the membrane was incubated with anti-ATF3 primary antibody at 4°C overnight and then incubated with horseradish peroxidase (HRP)-labeled secondary antibody at room temperature for 1 hour. Finally, Western blot was performed using enhanced chemiluminescence (ECL) substrates.

### Transwell assay

The Transwell assay (88) was employed to evaluate cell migration and invasion capabilities. First of all, the cells underwent a 24-hour incubation in serum-free culture medium. Transwell chambers, with or without Matrigel, were prepared, and a cell suspension was created. The lower chamber was filled with ECM containing serum, while serum-free ECM was used in the upper chamber. Cells were then added to the upper chamber and incubated for 48 hours. After incubation, cells were fixed using 4% paraformaldehyde (PFA) and then stained with crystal violet (Solarbio, China) for 10 minutes. Migrated cells on the underside of the membrane were counted under a microscope to assess cell migration or invasion.

### 5-Ethyl-2 ‘ -deoxyuridine proliferation assay

This assay enabled rapid detection of cellular DNA replication activity, providing further assessment of cell proliferation. Treated cells (5 × 10^3^ per well) were seeded into a 6-well plate and cultured for 24 hours. During cultivation, EDU was added to the medium for cellular uptake. The cells were cultured for 2 hours. Afterward, the EDU-containing medium was removed, and cells were washed with PBS 1–2 times for 5 minutes each. Cells were fixed with 50 μL of 4% paraformaldehyde for 30 minutes, treatment with 100 µL permeabilization buffer at room temperature for 10–15 minutes. The cells were then treated with glycine (2 mg/ml) and 0.5% Triton X-100 for 15 min and stained with 1X Apollo and 1X Hoechst for 30 min. A fluorescent dye was applied to visualize EDU labeling. Images of stained cells were captured using a fluorescence microscope or other suitable equipment.

### Tube formation assay

The tube formation assay (20) was primarily utilized to screen for anti-angiogenic or pro-angiogenic agents. HUVEC were cultured in ECM medium supplemented with 10% fetal bovine serum (Gibco BRL, USA) and 1% streptomycin/penicillin for 24 hours. Once cell confluency reached 80%, the original medium was replaced with ECM containing 0.2% FBS, 2 mM L-glutamine, 1 mM sodium pyruvate, 100 U/mL penicillin, and 100 μg/mL streptomycin, and incubation was continued for an additional 24 hours. Each well in a 96-well plate was coated with 100 μL of Matrigel and allowed to gel at 37°C for 30 minutes. Approximately 3 × 10^4^ cells were then seeded into each well, and the plate was incubated at 37°C with 5% CO_2_ and 95% humidity. After 16 hours, capillary-like structures were observed and imaged using an optical microscope.

### Statistical analysis

All figures and statistical analyses were conducted using R (version 4.3.0) and Python software (version3.7). Differences between groups were calculated using the Wilcoxon test and Pearson correlation coefficient (89). The criterion was set as *P < 0.05, **P < 0.01, ***P < 0.001, and ****P < 0.0001. ‘ns’ was considered not statistically significant.

## Results

### Characterization of different cell types in BAVM

**Figure 1:**
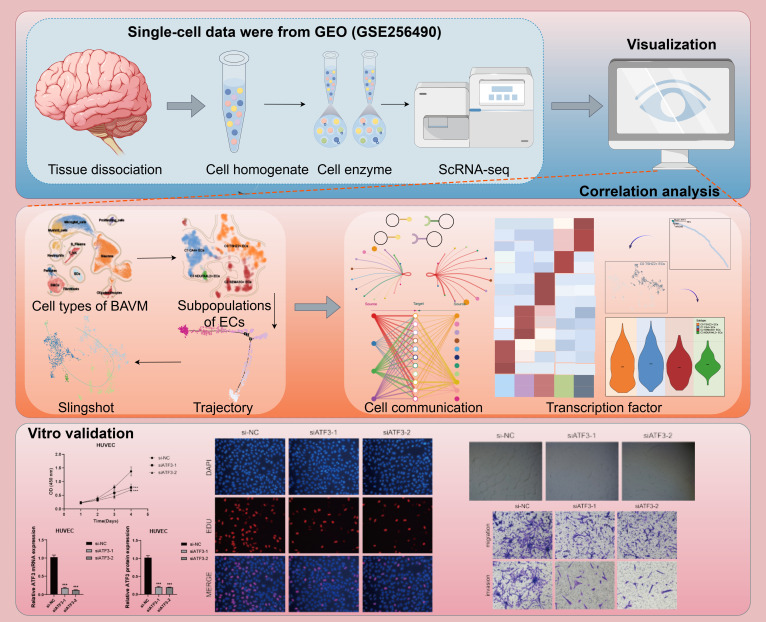
Flow chart of this study. Created using the Figdraw software. The image ID is STIUAaae93.

**Figure 2:**
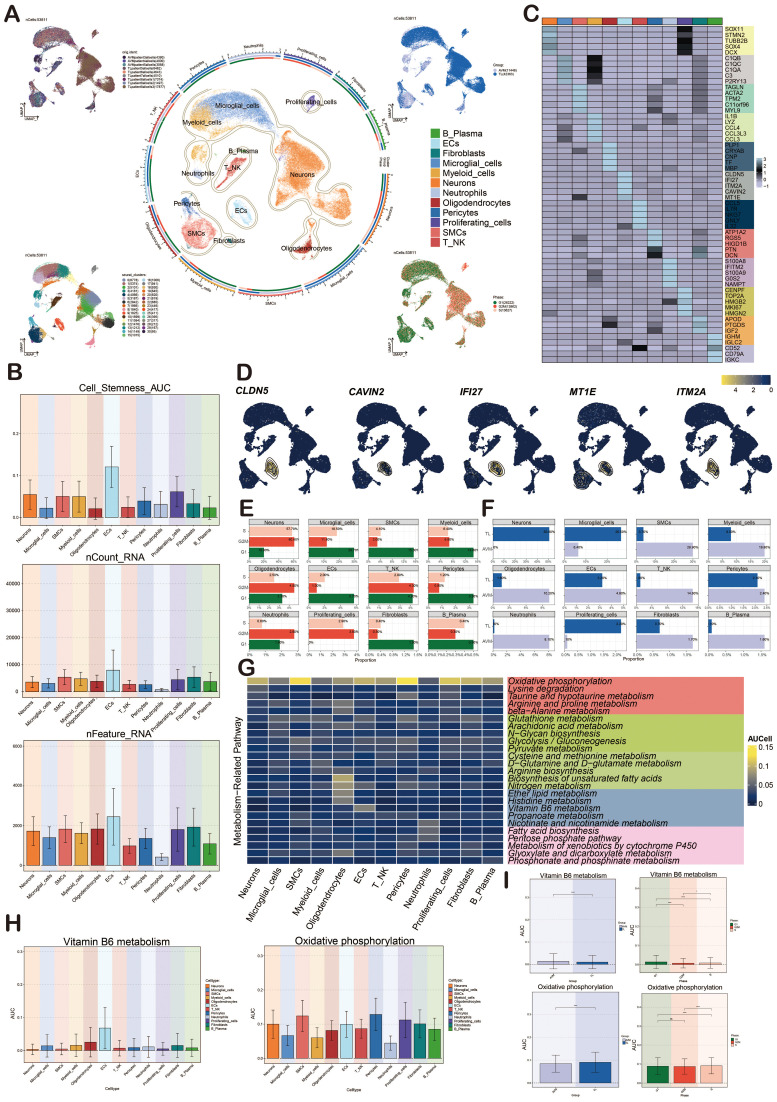
Characterization of cell types in BAVM by scRNA-seq technology. **(A)** The UMAP maps showed that BAVM cells were derived from 3 AVM patients and 6 TL patients (upper left). These cells were divided into 31 cell clusters (lower left) and 12 cell subsets (middle). The expression of these cells in different phases (lower right) and groups (upper right) was shown, and different cell clusters and cell types were represented by different colors. **(B)** The bar graphs depicted the nCount_RNA, nFeature_RNA, and Cell Stemness AUC for the 12 cell types, with different colors representing various cell types. **(C)** The heatmap demonstrated the differential expression of the top 5 marker genes among the 12 cell types, with the top 5 marker genes for ECs being *CLDN5, IFI27, ITM2A, CAVIN2*, and *MT1E*. **(D)** The UMAP diagrams displayed the expression levels of *CLDN5, IFI27, ITM2A, CAVIN2*, and *MT1E* across various cell types, with brighter colors indicating stronger expression. **(E)** The bar graphs illustrated the proportions of the 12 cell types within different cell cycles (G1, G2M, and S). **(F)** The bar graphs presented the proportions of the 12 cell types across different groups (TL, BAVM). **(G)** The heatmap depicted the metabolic pathways of the 12 cell types. Oxidative phosphorylation was highly expressed in all cell types, and the metabolic process of vitamin B6 was mainly highly expressed in ECs. **(H, I)** Bar graphs showcased the AUCell score of vitamin B6 metabolism and oxidative phosphorylation across cell types, groups and cell cycles, with p-values derived from a paired Wilcoxon test. *P < 0.05 and ****P < 0.0001. ns, no statistical significance.

**Figure 1** illustrated the comprehensive flow of this study. We re-analyzed the scRNA-seq data with the registration number GSE256490 and visualized the cells using the UMAP map.The samples included two groups, BAVM and TL, primarily derived from three BAVM patients and six TL patients. A total of 31 cell clusters were identified, which could be categorized into 12 cell types: proliferating cells, microglial cells, myeloid cells, neutrophils, B plasma cells, T NK cells, pericytes, smooth muscle cells (SMCs), fibroblasts, ECs, neurons cells, and oligodendrocytes. Then, the cells in the G2M and S phases were mostly distributed on the right side, mainly in the TL group, while the cells in the G1 phase were mostly concentrated on the left side, mainly in the BAVM group (**Figure 2A**). To understand the expression of various cell types in the samples, we used bar charts to display the nCount_RNA, nFeature_RNA, and Cell_Stemness_AUC for each cell types (**Figure 2B**). Notably, ECs had the highest expression levels, showing significant specificity, suggesting a potential link between highly expressed ECs and BAVM. A heatmap was used to show the top 5 marker genes of each subpopulation, with ECs marker genes being *CLDN5, IFI27, ITM2A, CAVIN2*, and *MT1E* (**Figure 2C**), which was further validated using UMAP plots (**Figure 2D**). Next, bar charts were used to display the proportion of each cell types in different cell cycles. We found that neurons had the highest proportion in the S phase (57.7%) and the G2M phase (60.6%). ECs accounted for 5.7% in the G1 phase, 1.0% in G2M, and 2.0% in the S phase (**Figure 2E**). Additionally, the proportion of different cell subpopulations in BAVM group and TL group was different. We found that neurons, microglial cells, and proliferating cells were primarily expressed in TL, while the remaining cell types were more expressed in BAVM (**Figure 2F**).

Cellular metabolic pathways could influence BAVM development. We found that oxidative phosphorylation was the most prominent metabolic pathway in all subpopulations. We found that the heatmap showed that the vitamin B6 metabolic pathway was mainly highly expressed in ECs, but no obvious specificity was found in other subpopulations (**Figure 2G**). The bar plots showed the AUCell score of oxidative phosphorylation and vitamin B6 metabolic pathways in various cell types, phases, and groups (**Figures 2H, I**). The results showed that these two metabolic pathways were highly expressed in ECs, especially the vitamin B6 metabolic pathway. However, their expression differences in different periods and groups were very small.

### Characterization of ECs heterogeneity in BAVM by scRNA-seq technology

**Figure 3:**
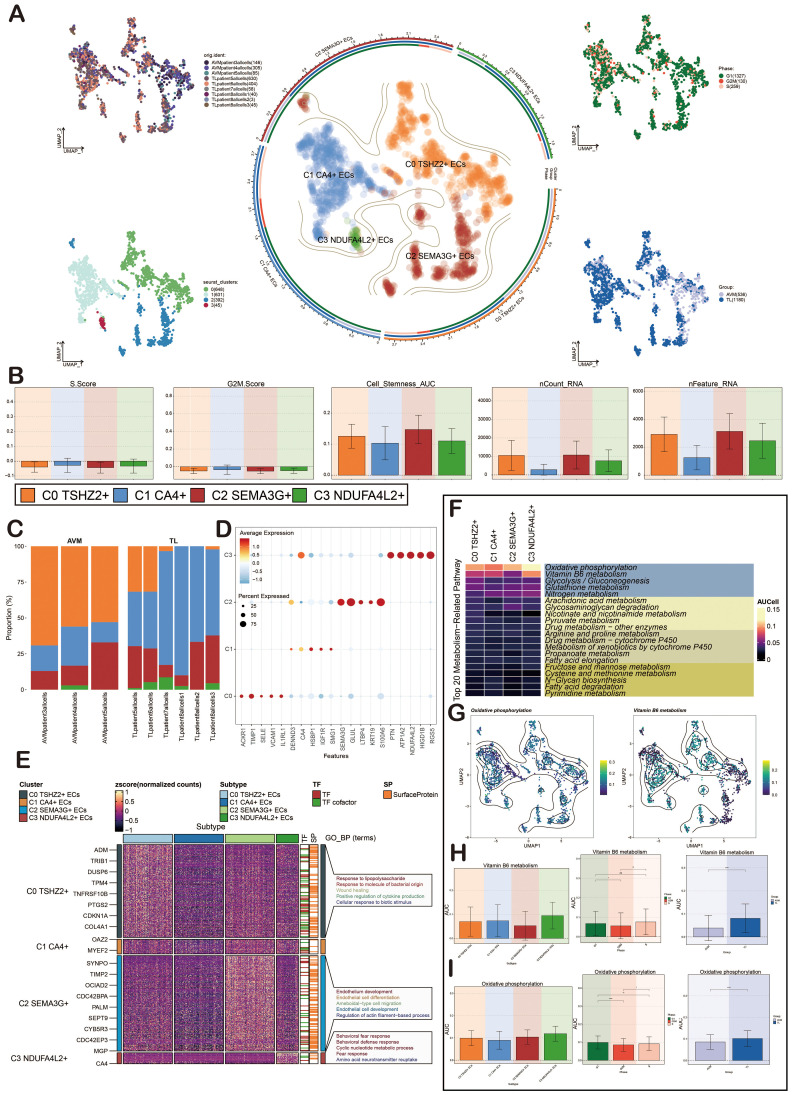
Characterization of ECs in BAVM by scRNA-seq technology. **(A)** The UMAP diagrams showed that the ECs in BAVM came from 3 AVM patients and 6 TL patients. The ECs were divided into 4 cell clusters (lower left) and 4 cell subpopulations (middle), and the expression of these cells in different phases (upper right) and groups (lower right) was shown. Each point represents one cell. **(B)** The bar diagrams illustrated the expression level of the four cell subpopulations in terms of nCount_RNA, nFeature_RNA, S.Score, G2M.Score, and Cell Stemness AUC. **(C)** The bar plots represented the proportion of the four EC subpopulations across the groups (TL, BAVM). **(D)** The bubble diagram illustrated the differential expression of the top 5 marker genes across various cell subpopulations, with bubble color based on standardized data and size representing the percentage of gene expression in the subpopulations. **(E)** The heatmap illustrated the biological processes that were significantly enriched in different cell subpopulations, as determined by GOBP enrichment analysis. **(F)** The heatmap illustrated the AUCell score of the top 20 metabolism-related pathways and AUCell in ECs, as depicted in the figure. **(G)** The UMAP diagrams represented the distribution of oxidative phosphorylation and vitamin B6 metabolism. **(H)** The bar diagrams depicted the AUCell score of vitamin B6 metabolism across EC subpopulations, groups, and cell cycles. *P < 0.05, **P < 0.01, and ****P < 0.0001. 'ns' was considered not statistically significant. **(I)** The bar diagrams showcased the AUCell score of oxidative phosphorylation within EC subpopulations, groups, and cell cycles. *P < 0.05, **P < 0.01, and ****P < 0.0001.

We conducted further analysis on ECs. Initially, UMAP plots showed that ECs were mainly from 9 patient samples. After preliminary quality control, we obtained 1,716 high-quality cells, which were organized into four distinct cell clusters. Based on specific gene expression markers, the ECs were categorized into four subpopulations: C0 *TSHZ2*+ ECs, C1 *CA4*+ ECs, C2 *SEMA3G*+ ECs, and C3 *NDUFA4L2*+ ECs. Subsequently, we depicted various cell cycles, observing that G2M and S phases were concentrated in the upper corner (**Figure 3A**).

To elucidate the differences among EC subpopulations, we used bar plots to illustrate the scores of nCount_RNA, nFeature_RNA, S.Score, G2M.Score, and Cell_Stemness_AUC across the four cell subpopulations. The results revealed that C0 *TSHZ2*+ ECs had elevated scores in nCount_RNA, nFeature_RNA, and Cell_Stemness_AUC, suggesting this subpopulation might exist in a highly active state (**Figure 3B**). The bar diagrams illustrated the proportionof these four cell subpopulations in BAVM and TL. It was evident that C0 *TSHZ2*+ ECs differed significantly between BAVM and TL, accounting for the largest proportion in BAVM and a small amount in TL (**Figure 3C**). This finding led us to hypothesize that this subpopulation may be implicated in the formation and progression of BAVM, thereby capturing our interest. The bubble diagrams displayed the top five marker genes within each EC subpopulation (**Figure 3D**). Then, a heatmap presented the enrichment analysis results of the four EC subpopulations, showcasing associated enrichment genes and pathways (**Figure 3E**). In the process of BAVM development, the Top 20 Metabolism-Related Pathways of ECs, among which the top five pathways include: oxidative phosphorylation, vitamin B6 metabolism, glycolysis/gluconeogenesis, glutathione metabolism, nitrogen metabolism, and glycolytic degradation. Oxidative phosphorylation was the most obvious in EC subpopulations (**Figure 3F**). We further demonstrated the AUCell score of these metabolic pathways by using the UMAP diagrams, especially glycolysis/gluconeogenesis and vitamin B6 metabolism (**Figure 3G**). The AUCell score of these metabolic pathways in the EC subpopulations, different groups, and phases were demonstrated by the bar diagrams. In both pathways, the results showed that the AUCell score was higher in C0 *TSHZ2* + ECs and C3 *NDUFA4L2*+ ECs, and higher in TL and G1 phase. (**Figures 3H, I**).

### CytoTRACE and Monocle2 analysis of ECs

**Figure 4:**
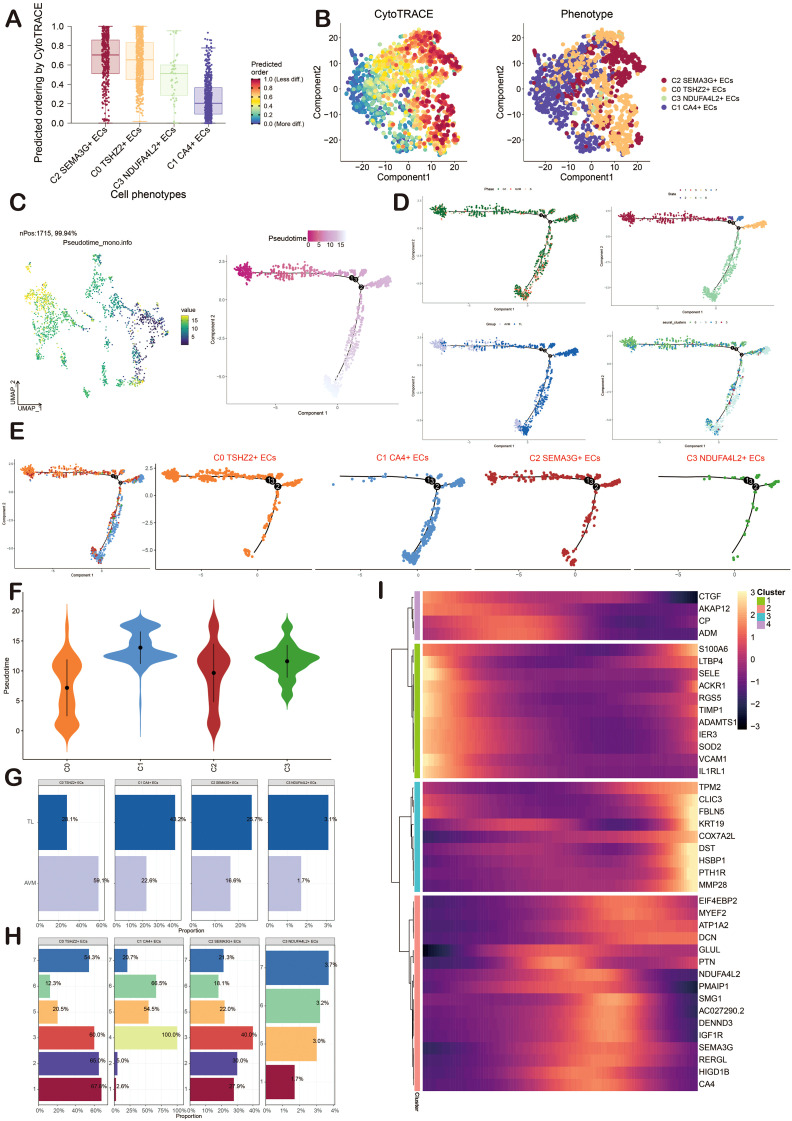
Visualization of CytoTRACE and Monocle2 analysis of ECs in BAVM. **(A)** The box chart illustrated the predicted differentiation of ECs as assessed by CytoTRACE, with the sequence from highest to lowest being C2, C0, C3, and C1. **(B)** The right image displayed the analysis of ECs differentiation via CytoTRACE, with different cell subpopulations represented by distinct colors in the figure. **(C)** The pseudotime trajectory graphs illustrated the overall pseudotime trajectory of ECs differentiation throughout the progression of BAVM. **(D, E)** The pseudotime trajectory graphs illustrated the distribution of cell cycles, states, groups, and EC subpopulations. **(F)** The violin diagram depicted the differentiation order among the four EC subpopulations. **(G, H)** The bar graphs presented the proportions of the four cell subpopulations across different groups (TL, BAVM) and states (states 1–7). **(I)** The heatmap displayed the dynamic trajectory of genes within each subpopulation.

CytoTRACE and Monocle2 were mainly used to reveal the stemness properties and differentiation trajectory of ECs. At first, we used CytoTRACE to analyze the differentiation ability of ECs and revealed the differentiation potential of cells. Then, the cell stemness of the four cell types was further displayed by the box chart, and the stemness from high to low was C2, C0, C3, and C1 (**Figures 4A, B**). Pseudotime analysis showed that BAVM differentiated from the upper left (**Figure 4C**). We also depicted the differentiation trajectory of ECs across phases, states, groups, and clusters throughout the progression of BAVM, with the results presented in the figure. The pseudotime sequence trajectorys of four cell subpopulations were also shown in the figure, starting from the upper left to the lower right. The could be clearly seen that C0 *TSHZ2*+ ECs were located at the starting point and gradually differentiated during the progress of BAVM (**Figures 4D, E**). The results showed that the C0 *TSHZ2*+ ECs accounted for the highest proportion in BAVM, and other subpopulations mainly accounted for the highest proportion in TL, which also proved that C0 *TSHZ2*+ ECs may be the key subpopulation in BAVM.

Then, the differentiation sequence of four cell types of ECs was shown by the violin diagram, and we found that the differentiation sequence was C0→C2→C3→C1 (**Figure 4F**). The bar graph also showed the proportion of four cell types of ECs in different groups (BAVM and TL) and different states (**Figures 4G, H**). We divided these cells into four subpopulations and showed the dynamic trajectory of genes in each subpopulation by heatmap (**Figure 4I**).

### Slingshot analysis of ECs

**Figure 5:**
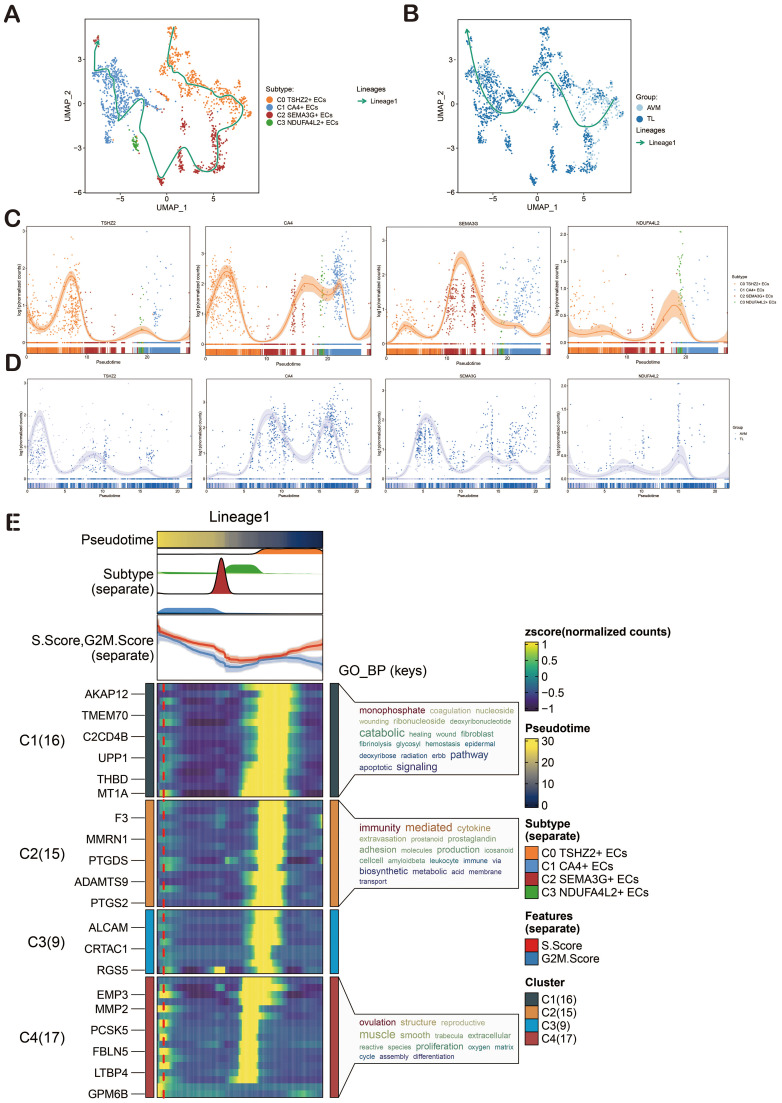
Slingshot Analysis of ECs on Pseudotime Trajectory. **(A)** The UMAP diagram illustrated the distribution of pseudotime trajectories among four cell types in lineage 1. **(B)** The UMAP diagram showcased the distribution of pseudotime trajectories among various groups in lineage 1. **(C)** The dynamic trend diagram showed the dynamic trajectory expression of the named genes (*TSHZ2, CA4, SEMA3G, NDUFA4L2*) of EC subpopulations on lineage 1. **(D)** The dynamic trend graph showed the expression of the named genes of EC subpopulations in different groups. **(E)** GO-BP enrichment analysis confirmed the biological processes associated with lineage 1 of ECs.

In order to infer the pseudotime trajectory of ECs, we used Slingshot to analyze it and get a differentiation trajectory: lineage 1. We found that C0 *TSHZ2*+ ECs was the starting point of lineage 1 differentiation, and the sequence was C0→C2→C3→C1. In addition, the change of groups in lineage 1 was also shown. The dynamic expression of each named gene in lineage 1 also clearly showed that *TSHZ2* was mainly at the initial point of the trajectory (**Figures 5A–D**). Then, the differentiation trajectory was visualized by GO-BP enrichment analysis (90, 91). It was found that C1 in lineage 1 was related to monophosphate, C2 was related to immunity and mediated, and C4 was related to ovulation (**Figure 5E**).

### CellChat analysis among cell subpopulations of BAVM

**Figure 6:**
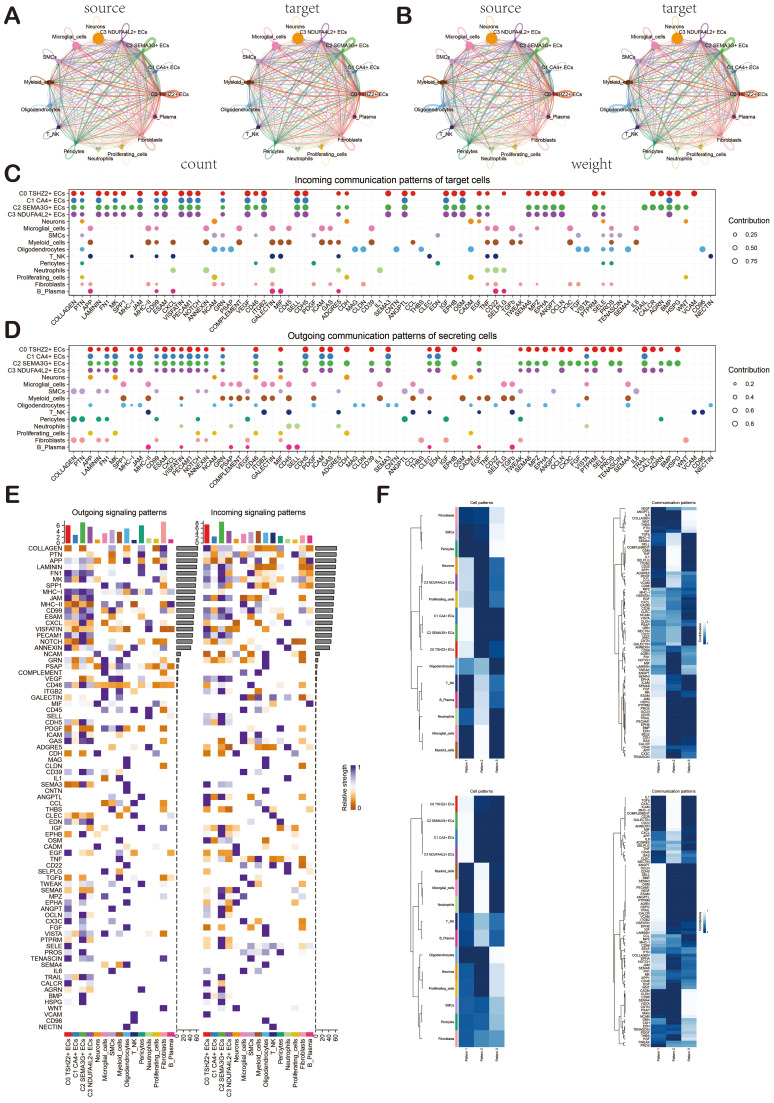
Cellchat analysis showed the interaction of cell types in BAVM. **(A, B)** Circular diagrams illustrated the number (left) and weight (right) of interactions among all cell types. **(C, D)** The bubble diagrams depicted the incoming communication patterns of target cells and the outgoing communication patterns of secreting cells. **(E)** The heatmap displayed the intensity of incoming and outgoing signaling pathways for all cell types, categorized into outgoing signaling patterns and incoming signaling patterns. **(F)** The heatmap illustrated the communication pattern recognition of the efferent cell (up) and the afferent cell (down) across all cell types.

To gain a deeper insight into cellular interactions, we employed CellChat analysis. First of all, we established the communication network between cells in BAVM, including proliferating cells, microglia cells, myeloid cells, neutrophils, B-plasma cells, T_NK cells, pericytes, SMCs, fibroblasts, ECs, neurons cells, and oligodendrocytes, and showed the count and weight of cell-to-cell interaction through cell interaction circle diagrams (**Figures 6A, B**). To identify the key incoming and outgoing signals associated with BAVM, we illustrated the incoming communication patterns of target cells and outgoing communication patterns of secret cells by bubble diagram. We further predicted their pivotal incoming and outgoing signals, exploring the progression of BAVM through ligand-receptor interactions (**Figures 6C, D**). In order to understand the signal pathways of all types of cells in BAVM. The incoming and outgoing signal pathways were analyzed by heatmap. In the incoming signal part, most ECs expressed the MK signaling pathway. In the signal outgoing part, mainly expressed in C0 and C2 subpopulations (**Figure 6E**). Therefore, we speculated that MK was closely related to ECs and BAVM. Finally, we explored how cells and signaling pathways interacted by analyzing gene expression patterns. First of all, the heatmap showed cell patterns and communication patterns and corresponded them one by one, which were divided into pattern 1, pattern 2, and pattern 3. The upper part was the output signal, and the lower part was the input signal. ECs were mainly expressed in pattern 1 of the outgoing signal and were closely related to signaling pathways such as ANGPT, OCLN, and CDH5. Myeloid cells and microglia were mainly expressed in pattern 2. Oligodendrocytes are mainly expressed in pattern 3. In addition, the content of the incoming signal section was shown in the figure (**Figure 6F**).

### ECs signal pathway MK and ligand-receptor pair MDK-NCL

**Figure 7:**
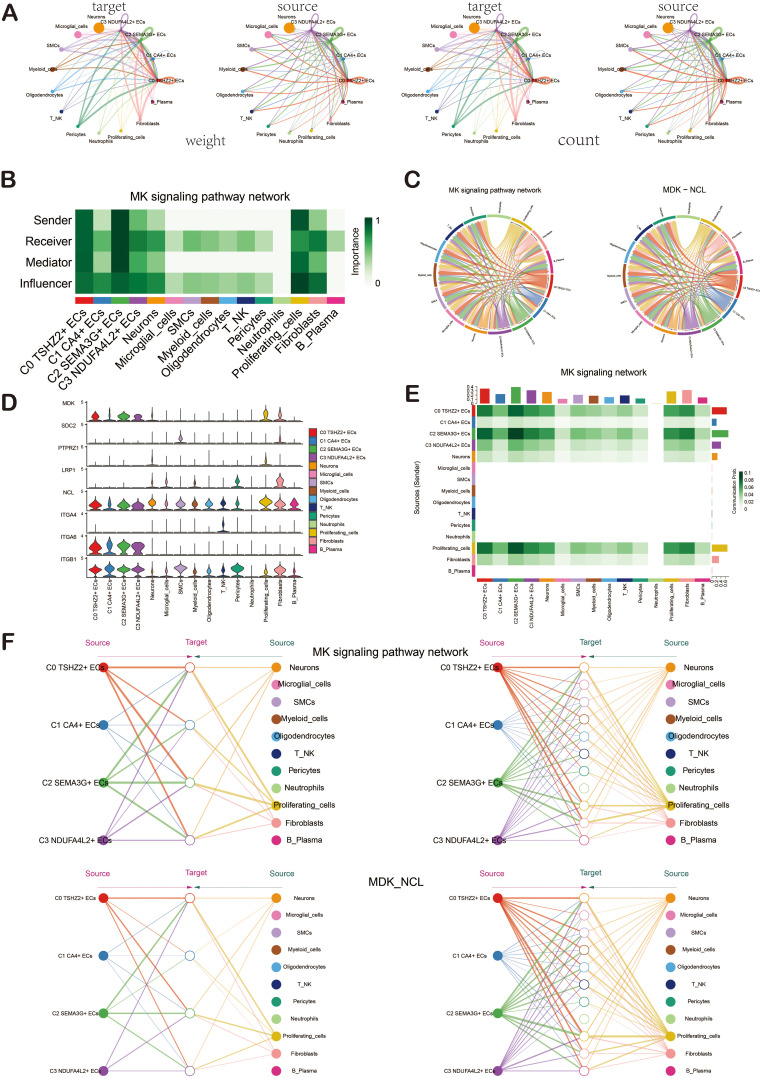
Cellchat analysis showed the interaction of ECs. **(A)** The circular diagrams illustrated the weight (left) and count (right) of interactions between ECs and other cell types. **(B)** The heatmap displayed the centrality scores of the MK signaling pathway network, highlighting the varying importance of different cell subpopulations. **(C)** The chord diagrams depicted the cellular interactions within the MK signaling pathway network, as well as those involving the MDK-NCL ligand-receptor pair. **(D)** The violin diagram illustrated the cellular interactions associated with the MK signaling pathway. **(E)** The heatmap represented the cellular interactions within the MK signaling pathway. **(F)** Hierarchical diagrams showcased the interactions among various cell types in the MK signaling pathway network (top) and the MDK-NCL ligand-receptor pair (bottom).

In order to explore the action mode of the MK signaling pathway and its related ligand-receptor pairs, we made a visual analysis of them. We first showed the cell communication between ECs and other cell types, including the weight and count of cell interaction. Among them, ECs were closely related to various cells (**Figure 7A**). Then, through the centrality measurement, we determined the centrality score of the MK signaling pathway network (**Figure 7B**). According to the chord diagrams, the cell interaction was in the MDK signaling pathway and the MDK-NCL ligand-receptor pair (**Figure 7C**). The violin diagram also confirmed that C0 *TSHZ2*+ ECs was highly expressed in MDK, and NCL was expressed in almost all cell types of BAVM (**Figure 7D**). The interaction between cells in the MK signaling pathway was shown in the figure, which was mainly related to C0 *TSHZ2*+ ECs and C2 *SEMA3G*+ ECs (**Figure 7E**). The hierarchical diagram of the interaction between ECs and other cells in the MK signaling pathway and the MDK-NCL ligand-receptor pair was shown in the figure (**Figure 7F**).

### Gene regulatory network analysis of ECs in BAVM

**Figure 8:**
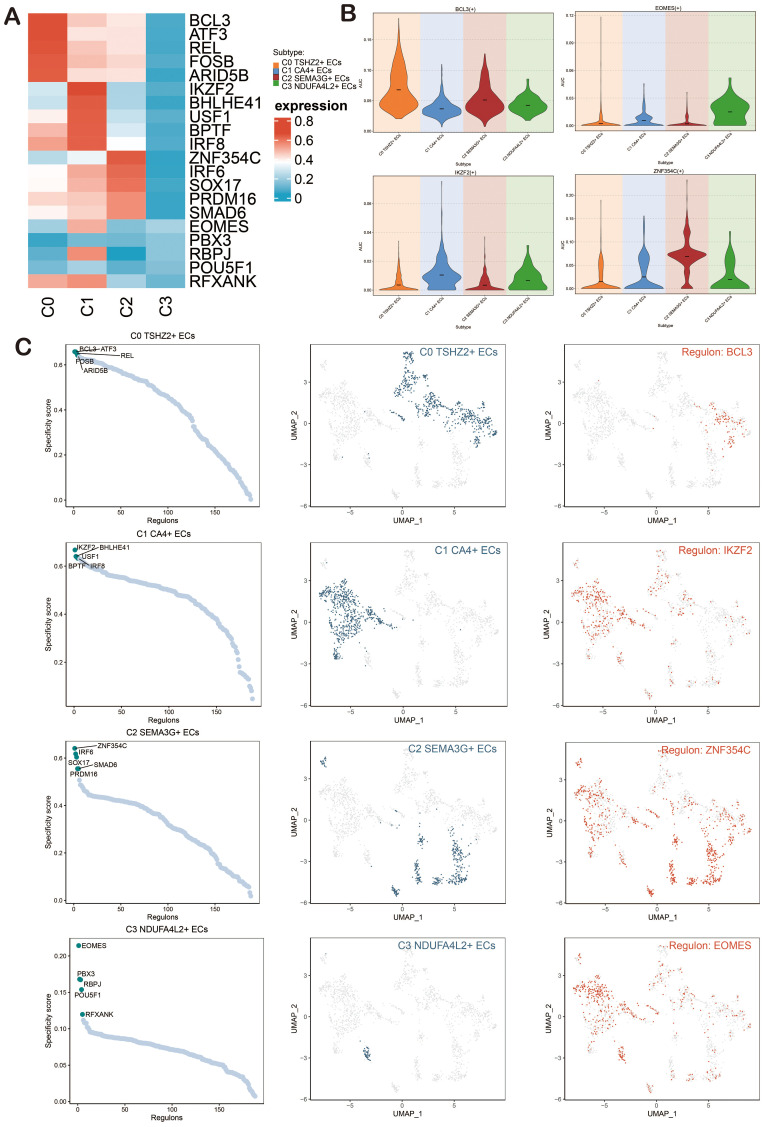
Analysis of Gene Regulatory Networks in ECs of BAVM. **(A)** A heatmap depicted the expression levels of the top-ranked TFs across four EC subpopulations, with red indicating high expression and blue indicating low expression. **(B)** Violin plots illustrated the expression of the active regulatory factors, including *BCL3, IKZF2, ZNF354C*, and *EOMES*, within EC subpopulations. **(C)** The scatter plots presented the ranking of regulatory factors in EC subpopulations of BAVM based on the RSS). The UMAP plots displayed the distribution of ECs (green dots), while the highest-ranked regulators in the EC subpopulations were highlighted in red on the UMAP.

To investigate the enrichment of key TFs and the activity of regulatory factors in ECs, we inferred the corresponding gene regulatory network using pySCENIC. Initially, we employed a heatmap to visualize the expression levels of the top-ranked TFs across each EC subpopulation, emphasizing the most active TFs: BCL3 (C0 *TSHZ2*+ ECs), IKZF2 (C1 *CA4*+ ECs), ZNF354C (C2 *SEMA3G*+ ECs), and EOMES (C3 *NDUFA4L2*+ ECs) (**Figure 8A**). The violin charts were used for a more intuitive display of the AUCell scores of BCL3, IKZF2, ZNF354C, and EOMES across different EC subpopulations (**Figure 8B**). The regulatory factors were ranked based on the Regulatory Specificity Score (RSS). In the UMAP plots, the EC subpopulations of BAVM were highlighted (red dots), followed by a display of the binarized Regulator Activity Score (RAS) of major regulators in each EC subpopulation (green dots) to validate the findings (**Figure 8C**).

**Figure 9:**
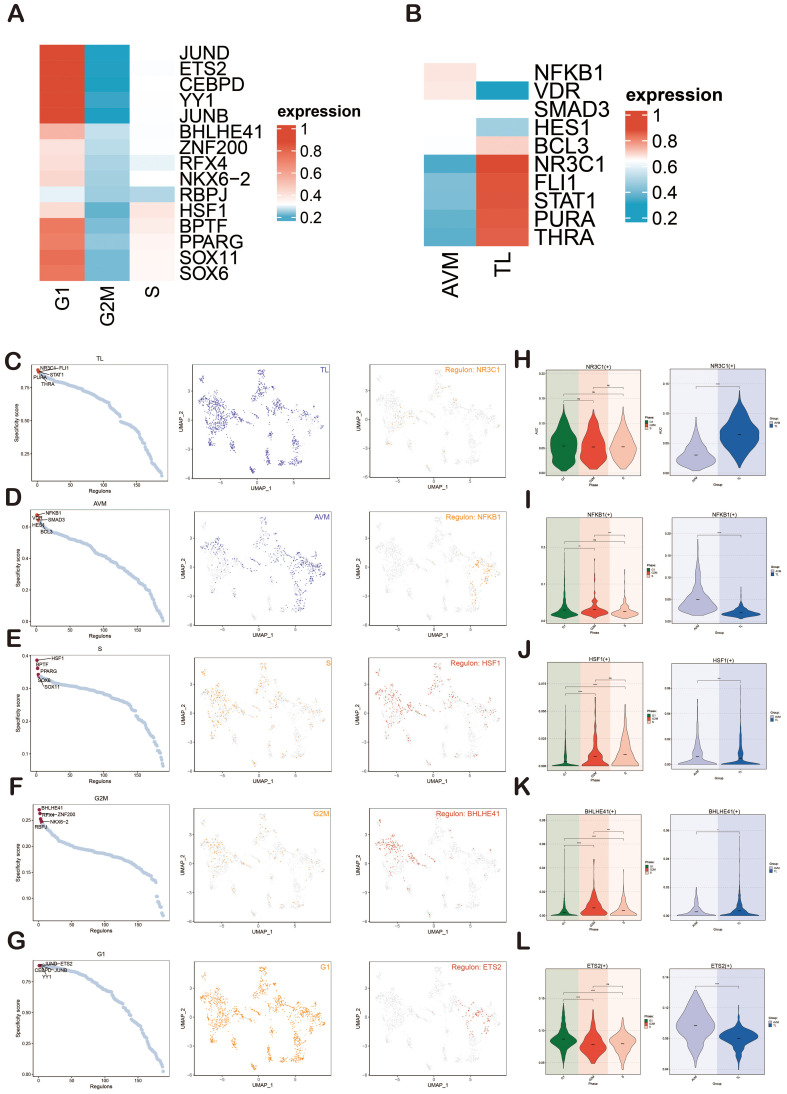
Gene regulatory network analysis of group and phase. **(A, B)** The heatmaps depicted the expression of the top-ranked TFs across various groups **(A)** and cell cycles **(B)**, with red indicating high expression and blue denoting low expression. **(C–G)** Scatter plots illustrated the rankings of regulatory factors across different groups and cell cycles based on the RSS. UMAP plots displayed the distribution of various groups and cell cycles (represented by red dots), with the top regulatory factors highlighted as green dots on the UMAP. **(H–L)** Violin plots revealed the expression of the most active regulatory factors, including NR3C1 (TL), NFKB1 (BAVM), HSF1 (S), BHLHE41 (G2M), and ETS2 (G1), within ECs across different groups and cell cycles. *p < 0.05, **p < 0.01, ***p < 0.001, ****p < 0.0001, ns indicated no statistical significance.

Next, we presented heatmaps showing the expression levels of the top-ranked TFs in different cell cycles (G1, G2M, S) and groups (TL, BAVM) (**Figures 9A, B**). The most active TFs were identified, including NR3C1 (TL), NFKB1 (BAVM), HSF1 (S), BHLHE41 (G2M), and ETS2 (G1) (**Figures 9C-G**). To compare the differences in these TFs across different phases and groups, we used violin plots. NR3C1 was highly expressed in G1, G2M, and S phases, with higher expression in TL. NFKB1 was primarily expressed in the G1 and G2M phases, with higher expression in BAVM. HSF1 showed higher expression in the S and G2M phases and higher expression in BAVM. BHLHE41 had higher expression in the S and G2M phases and was expressed highly in both TL and BAVM. ETS2 was prominently expressed in the G1 phase, with higher expression in BAVM (**Figures 9H-L**).

### Identification of gene regulatory modules in BAVM

**Figure 10:**
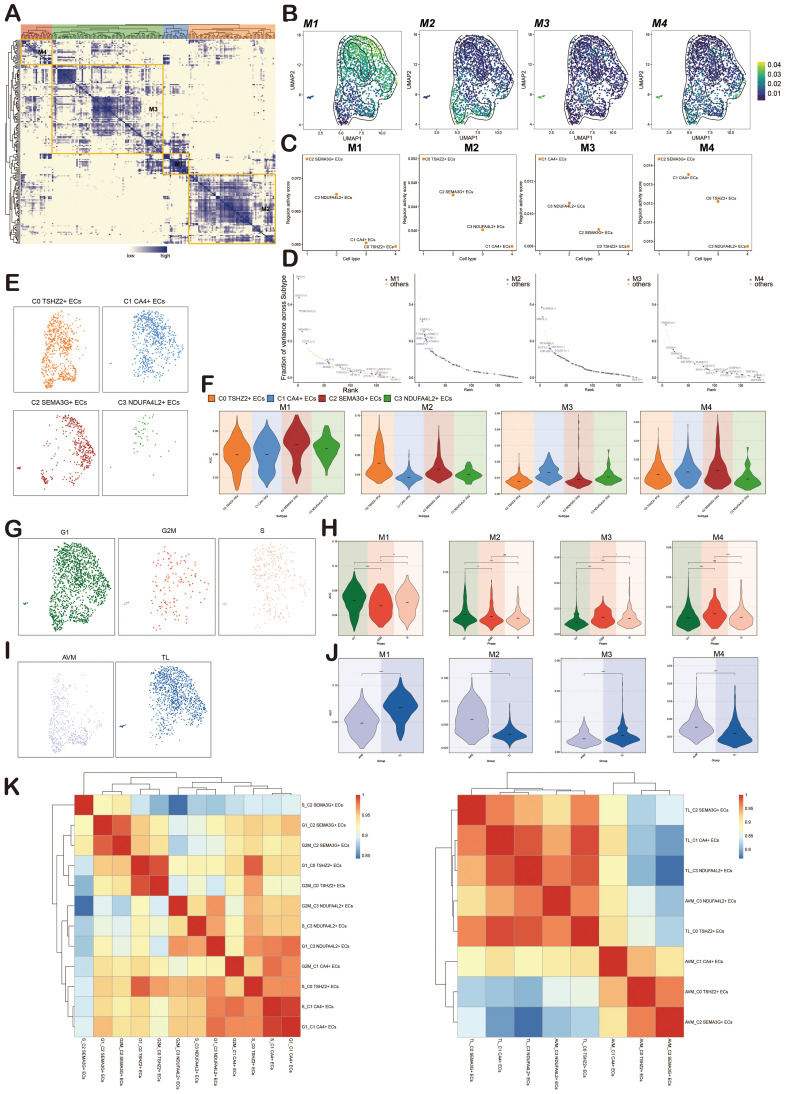
Identification of Regulatory Modules of TFs in BAVM. **(A)** A heatmap revealed four EC subpopulations-specific regulatory modules (M1, M2, M3, M4) identified based on the CSI matrix. **(B)** UMAP plots illustrated the distribution of average AUCell scores across M1, M2, M3, and M4. **(C)** Scatter plots depicted the regulatory activity scores of each EC subpopulation within M1, M2, M3, and M4. **(D)** Scatter plots ranked the key tfs within M1, M2, M3, and M4. **(E)** Facet plots illustrated the distribution of EC subpopulations in M1, M2, M3, and M4. **(F)** Violin plots displayed the expression of EC subpopulations within M1, M2, M3, and M4. **(G)** Facet plots showed the distribution of different cell cycles (G1, G2M, S) in M1, M2, M3, and M4. **(H)** Violin plots illustrated the expression of different cell cycles within M1, M2, M3, and M4. *p < 0.05, **p < 0.01, and ****p < 0.0001, ns indicated no statistical significance. **(I)** Facet plots depicted the distribution of different groups (TL, BAVM) within M1, M2, M3, and M4. **(J)** Violin plots displayed the expression of different groups within M1, M2, M3, and M4. ****p < 0.0001. **(K)** The heatmaps showed the correlation of transcriptional regulatory activity between EC subpopulations in different cell cycles (left) and different groups (right).

We used pySCENIC to determine the regulatory modules in ECs of BAVM based on the connection-specific index (CSI) matrix. These were further categorized into four major modules, M1, M2, M3, and M4, based on the similarity of AUCell scoring patterns (**Figure 10A**). Typical TFs and cell types were selected based on the average activity scores of each module. These TFs exhibited highly similar functions in one or several cell types. When the average activity scores of different modules were mapped onto the UMAP plot, the results showed clear differences in the cell subpopulations occupied by each module (**Figure 10B**).

TFs in M1 mainly regulated C2 *SEMA3G*+ ECs, TFs in M2 mainly regulated C0 *TSHZ2*+ ECs, TFs in M3 mainly regulated C1 *CA4*+ ECs, and TFs in M4 mainly regulated C2 *SEMA3G*+ ECs (**Figure 10C**). We then used scatter plots to show the key TFs in each module, finding that M1 was dominated by IRF6, SOX17, and ZNF354C, M2 by JUND, CEBPD, and ETS2, M3 by EOMES, RBPJ, and RFXANK, and M4 by PRDM16, SOX8, and RARG (**Figure 10D**).

To more intuitively show the expression of each EC subpopulation in M1, M2, M3, and M4, we used facet plots (**Figure 10E**) and violin plots (**Figure 10F**) for validation. Additionally, we displayed the expression of phases (G1, G2M, S) and groups (TL, BAVM) in M1, M2, M3, and M4 using facet plots and violin plots. We found that M1 and M2 were predominantly expressed in the G1 phase, while M3 had higher expression in the S and G2M phases, and M4 showed the most significant expression in the G2M phase. M2 and M4 were primarily expressed in BAVM, while TL expression was slightly higher in M1 and M3 compared to BAVM (**Figures 10G-J**). Finally, to understand the correlation of transcriptional regulatory activity between EC subpopulations in BAVM, we distinguished the EC subpopulations by different groups and cell cycles. From the heatmap, we observed high correlations between S_C2 *SEMA3G*+ ECs and G2M_C3 *NDUFA4L2*+ ECs, G2M_C3 *NDUFA4L2*+ ECs and S_C2 *SEMA3G*+ ECs, and others (**Figures 10K**, left). Additionally, the correlation between EC subpopulations in different groups also exhibited significant specificity (**Figures 10 K**, right).

### Experimental analysis

**Figure 11:**
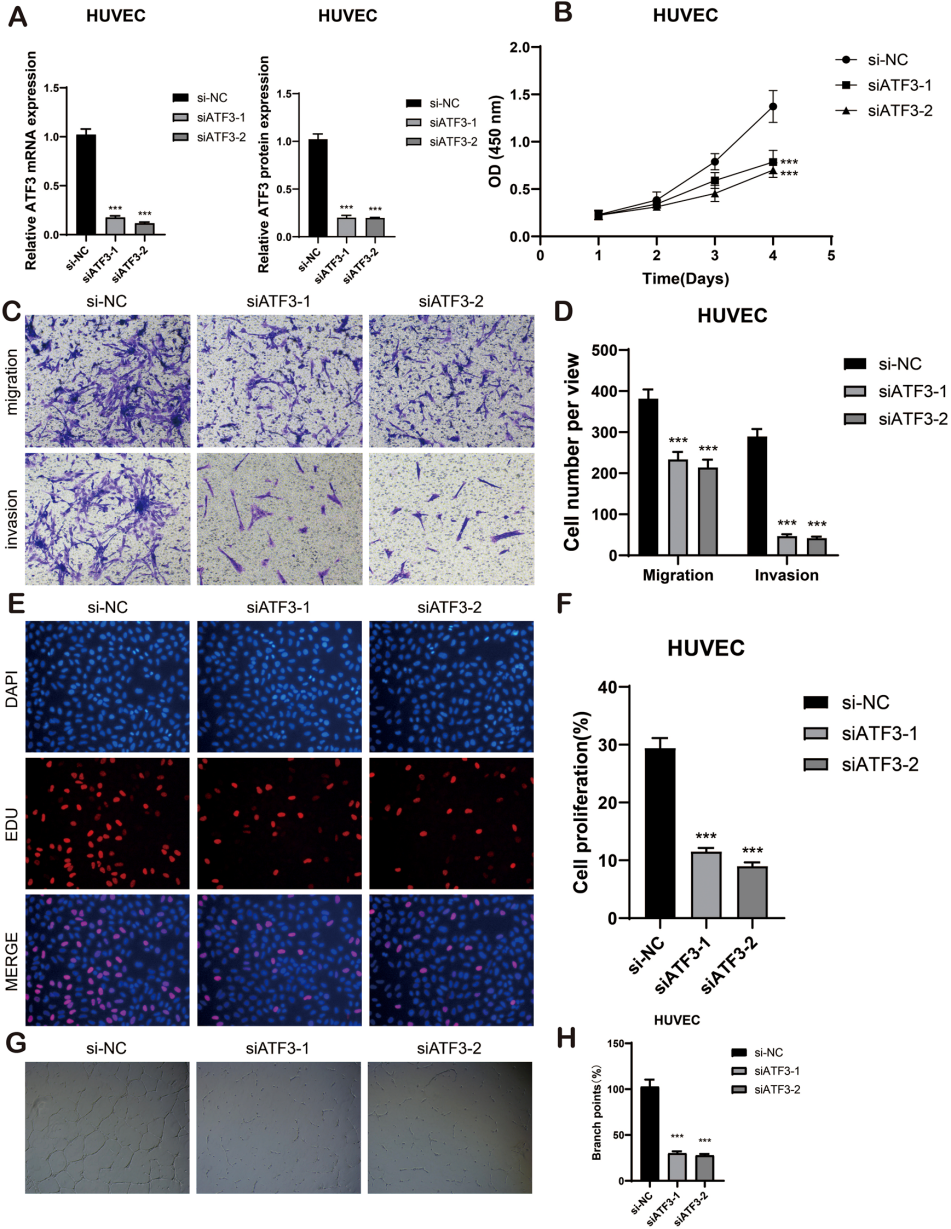
*In vitro* cell experiment. **(A)** The bar graphs showed the mRNA and protein expression of si-NC, siATF3–1 and siATF3–2 in HUVEC cell lines. ***p < 0.001. **(B)** The CCK-8 assay elucidated that the cell viability of the HUVEC cell lines experienced a significant decline subsequent to ATF3 knockdown. **(C, D)** The transwell assay demonstrated that, in comparison to the NC group, the migration and invasion capabilities of the HUVEC cell lines were substantially diminished following ATF3 knockout, ***p < 0.001. **(E, F)** The EdU staining assay disclosed that, in contrast to the NC group, ATF3 knockdown inhibited the proliferation of the HUVEC cell lines. ***p < 0.001. **(G, H)** Representative images from the tube formation assay illustrated a significant reduction in angiogenic capacity within the HUVEC cell lines after ATF3 knockout, as compared to the NC group. ***p < 0.001.

To further elucidate the role of ATF3 in BAVM, we conducted *in vitro* experiments primarily aimed at its function in ECs. The analysis was carried out to detect variations in ATF3 mRNA expression and protein expression within the HUVEC cell lines, revealing the expression levels of si-ATF3–1 and si-ATF3–2 in this context (**Figure 11A**).

The CCK-8 assay demonstrated a significant reduction in viability in HUVEC cell lines following ATF3 knockdown (P < 0.001) (**Figure 11B**) Transwell assays revealed that the knockdown of ATF3 significantly suppressed the migration and invasion abilities of HUVEC cell lines (**Figures 11C, D**). The EDU staining assay further confirmed that ATF3 knockdown suppressed cell proliferation (**Figures 11E, F**). In the tube formation assay, we observed that, compared to si-NC, cells treated with si-ATF3–1 and si-ATF3–2 exhibited reduced proliferation and impaired angiogenic capacity (**Figures 11G, H**). All experiments were conducted using HUVEC cell lines, with statistical significance denoted as p < 0.05. Taken together, these results underscore the positive role of ATF3 in ECs proliferation and angiogenesis.

## Discussion

BAVM is traditionally recognized as a congenital cerebrovascular malformation, but recent studies have shown that it may have acquired characteristics. The pathological feature is the direct connection between the cerebral arteries and veins, and the capillary network is short-circuited. Such structural abnormalities can lead to severe clinical symptoms, including cerebral hemorrhage, ischemia, epilepsy, and neurological deficits (92). The annual rupture risk of BAVM is approximately 2%, and in the event of rupture, the risk of re-rupture increases fivefold. It primarily affects young and middle-aged adults, with the median age of onset between 20 and 40 years. Current therapeutic approaches primarily involve surgical resection, endovascular embolization, and stereotactic radiosurgery. However, no effective pharmaceutical treatments have been developed. Therefore, understanding the mechanisms underlying the progression of BAVM and identifying potential therapeutic targets is crucial.

With the development of medicine, treatment strategies continue to emerge (93–95). To develop safer and more effective treatment methods to guide clinical practice, We analyzed the samples of BAVM patients, focusing on the transcriptome characteristics of ECs in BAVM. We first divided the patient sample into 12 cell types, among which ECs were the highest expressed in Cell Stemness AUC, indicating that they may have high differentiation potential in BAVM. To further investigate this, we analyzed the ECs at a deeper single-cell level. After quality control, we identified 1716 high-quality cells, which were divided into four subpopulations based on specific gene expression markers: C0 *TSHZ2*+ ECs, C1 *CA4*+ ECs, C2 *SEMA3G*+ ECs, and C3 *NDUFA4L2*+ ECs. C0 *TSHZ2* + ECs scored significantly higher in nCount RNA, nFeature RNA, and Cell Stemness AUC, indicating that they were in a highly active state and were closely related to the occurrence and development of BAVM. Furthermore, C0 *TSHZ2*+ ECs represented the largest proportion of ECs in BAVM but were minimally present in control tissue. These findings suggest a potential role for C0 *TSHZ2*+ ECs in the development of BAVM. CytoTRACE and monocle analysis showed that C0 *TSHZ2*+ ECs were in the early stage of differentiation and predicted high stemness, suggesting that they had strong proliferation ability. In the Slingshot, Lineage1 represents the differentiation trajectory of ECs, and C0 *TSHZ2*+ ECs are also in the initial stage of the differentiation trajectory. All these indicate that it may play an important role in the progression of BAVM. Next, we used CellChat (96) analyze the interactions among the cell subpopulations of BAVM and visualized the signaling pathways of different cell types. The MK signaling pathway network revealed significant interactions between EC subpopulations and other cell types. The analysis of incoming and outgoing signaling pathways, represented by a heatmap, showed that most BAVM subpopulations expressed the MK pathway in the incoming signals, while the outgoing signals were predominantly expressed by the C0 subpopulation. Furthermore, we observed that the MDK-NCL ligand-receptor pair could play a role in BAVM progression. Previous studies have shown that MK (97, 98), a heparin-binding growth factor, can influence the development and advance of BAVM by affecting ECs. Studies have shown that MK overexpression can promote ECs growth to increase cell angiogenesis activity (99, 100). Furthermore, MK is also involved in various biological processes, including cell growth, proliferation, migration, and so on. Nucleolin (NCL) is a multifunctional protein that may indirectly contribute to cell proliferation, growth, and angiogenesis (101). Based on these findings, we hypothesize that the MK signaling pathway, along with the MDK-NCL ligand-receptor pair, could potentially promote EC proliferation and angiogenesis in BAVM. In the future, we would continue to explore, dig deeper, and verify key signaling pathways and their functions through experiments.In this study, we performed gene regulatory network analysis to identify key TFs involved in BAVM. The results from the CSI analysis revealed four main modules within the EC subpopulation: M1, M2, M3, and M4, with C0 *TSHZ2*+ ECs predominantly expressing genes in the M2 module. We also identified key TFs within the EC subpopulation, with ATF3 emerging as a critical TF in C0 *TSHZ2*+ ECs. ATF3 (102, 103), a member of the ATF/ccAMP effector element binding protein family, is known for its broad role in regulating transcription. It is involved in various biological processes, including the cell cycle, immune regulation, endocrine regulation, and tumorigenesis. Studies have shown that ATF3 plays an important role in driving regeneration, which is closely related to endothelial proliferation and repair of aorta (104) and activation of lung ECs proliferation and repair of pulmonary capillary regeneration after injury (103, 105–107). ATF3 plays a role in the formation and proliferation of ECs. Based on these findings, we hypothesized that ATF3 may contribute to angiogenesis by promoting ECs proliferation, potentially leading to the development of BAVM. To test this hypothesis, we conducted cell experiments. The results showed that inhibiting ATF3 could reduce the growth and migration of ECs, which confirmed the key role of ATF3 in BAVM. The HUVEC cell line was used in the experiment, which can effectively reflect the basic biological characteristics of ECs. Although HUVEC cannot fully simulate the pathological features of ECs in BAVM, previous studies have shown that this model has important value in revealing the regulation mechanism of ECs in BAVM (108, 109). However, it is worth noting that HUVEC, as a normal EC, is significantly different from bAVM lesion endothelium in terms of phenotypic characteristics (6). In order to overcome this limitation, we will use patient-derived diseased ECs for more in-depth verification in future studies. Of course, it must be noted that ATF3, as a stress-responsive gene, may change its function due to cell type and environmental changes. Limited by the inability to obtain detailed clinical information of the original data, including KRAS mutation status, whether the lesion is ruptured, patient gender and age, etc., it may limit the exploration of ATF3 function. In the future, we also will start from this aspect and carry out clinical sample verification (collecting surgically resected bAVM samples, detecting ATF3 protein expression, and performing correlation analysis with KRAS mutation status and clinical history) and *in vitro* experiments (constructing a KRAS mutant endothelial cell model or detecting ATF3 dynamic expression under controlled hypoxia/mechanical stress conditions).

In addition, in the metabolic pathway of BAVM, we observed that the oxidative phosphorylation metabolic pathway showed high scores in all EC subpopulations. Studies have shown that oxidative phosphorylation, a biological process occurring in mitochondria, refers to the oxidation of organic substances (including sugars, lipids, and proteins) *in vivo* to release energy and synthesize ATP (110), among which C0 *TSHZ2*+ ECs has the highest score, which proves that C0 *TSHZ2*+ ECs can greatly meet the energy demand of EC subpopulations, thus affecting the proliferation of ECs. In addition, oxidative phosphorylation is also related to the production of reactive oxygen species (ROS), and ROS activation will promote abnormal vascular proliferation (93). The oxidative phosphorylation pathway was highly expressed in C0 *TSHZ2* + ECs, so we could reasonably speculate that the increased ROS activation in C0 *TSHZ2* + ECs was one of the important reasons for the abnormal proliferation and angiogenesis of ECs promoted by this subpopulation. We also found that vitamin B6 metabolism was expressed in all four EC subpopulations, so we speculated that it was related to ECs.

Based on these, we proposed that C0 *TSHZ2*+ ECs played a pivotal role in the initiation and progression of BAVM. This study offered the first comprehensive characterization of ECs at the single-cell level, uncovering the disease’s molecular mechanisms. It makes up for the blank of ATF3 acting on BAVM, and provides a promising way to identify potential therapeutic targets and open up targeted therapy for BAVM.

However, the sample size of scRNA-seq in this study is small, which may lead to deviations in sequencing results, and there were still limitations in the study of metabolic pathways and signaling pathways. In the future, we will collect more samples to further verify these findings and explore their clinical application in order to promote precision medicine. Through the implementation of precise targeted therapy, it is expected to substantially improve the prognosis and quality of life of BAVM patients and lay a solid foundation for the creation of personalized treatment programs.

## Conclusion

BAVM is a common congenital cerebrovascular malformation, which may lead to recurrent intracranial hemorrhage, cerebral ischemia, seizures, and other neurological complications. Although there are surgical and endovascular treatment methods, drug treatment is still lagging behind. Therefore, it is particularly important to promote the clinical research on the pathogenesis and treatment strategies of BAVM. scRNA-seq and multi-omics studies play a key role in the progression of BAVM. ATF3 may be a potential target for inhibiting EC proliferation, providing a new direction for targeted therapy of BAVM. In the future, the effectiveness of ATF3 as a therapeutic target should be further verified, and its application in other vascular diseases should be explored to promote the development of precision medicine.

## Data Availability

The original contributions presented in the study are included in the article/**Supplementary Material**. Further inquiries can be directed to the corresponding author.
