# Supplementary material for: The role of ATF3 in precision medicine of brain arteriovenous malformation: based on endothelial cell proliferation
Source: Front Immunol. 2025 Jun 27;16:1567970. doi: 10.3389/fimmu.2025.1567970 (PMC12248220; doi:10.3389/fimmu.2025.1567970)
Supplement: Supplementary file 1 [file Table1.docx]

siRNA序列：

si-1：GCCUUUCAUCUGGAUUCUA

si-2：AUUUGAUAUACAUGCUCAA

qPCR序列：

F：AGCCATTGGAGAGCTGTCTT

R：AATGGCCAGTGTGTTAAGGC
